# Targeting the Undruggable: Recent Progress in PROTAC-Induced Transcription Factor Degradation

**DOI:** 10.3390/cancers17111871

**Published:** 2025-06-03

**Authors:** Hyein Jung, Yeongju Lee

**Affiliations:** Department of Chemistry, Pusan National University, Busan 46241, Republic of Korea; haein7052@pusan.ac.kr

**Keywords:** transcription factor, PROTAC, BRD4, AR, ERα

## Abstract

Transcription factors (TFs) play central roles in gene regulation and disease progression but have long been considered undruggable due to the absence of well-defined binding pockets and their reliance on protein–protein or protein–DNA interactions. Proteolysis-targeting chimeras (PROTACs) offer a novel strategy to overcome these limitations by inducing selective degradation of TFs via the ubiquitin–proteasome system. This review highlights recent advances in TF-targeting PROTACs, focusing on key oncogenic TFs such as androgen receptor (AR), estrogen receptor alpha (ERα), BRD4, c-Myc, and STAT family members. Strategies for ligand design—including small molecules, peptides, and nucleic acid-based elements—are discussed alongside the use of various E3 ligases such as VHL, CRBN, and IAP. Several clinically advanced PROTACs, including ARV-110 and ARV-471, demonstrate the therapeutic potential of this technology. Despite challenges in pharmacokinetics and E3 ligase selection, emerging data suggest that PROTACs can successfully target TFs, paving the way for new treatment strategies across oncology and other disease areas.

## Simple Summary

This manuscript reviews recent advancements in the development of PROTACs (proteolysis-targeting chimeras) designed to target transcription factors (TFs), which have been considered an undruggable target. The review outlines various TF ligands including small molecules, peptides, aptamers, and oligonucleotides, as well as the use of diverse E3 ligases like VHL, CRBN, MDM2, and IAP. This paper highlights the future direction of PROTACs targeting undruggable targets.

## 1. Introduction

**Figure 1:**
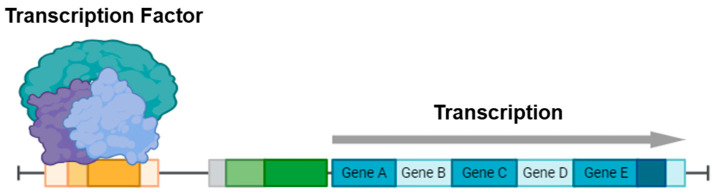
The schematic diagram of the mechanism by which transcription factors regulate the transcription of specific genes.

Transcription factors (TFs) are proteins that directly bind to DNA and initiate transcription, thereby regulating gene expression (Figure 1). As transcription is the first essential step in protein synthesis, TFs play a central role in this process and are often considered molecular “switches” that control gene expression. Accordingly, directly targeting TFs represents a promising strategy for suppressing aberrant gene expression in various diseases, including cancer. In particular, dysregulation of TF activity is frequently observed in oncogenesis, where the overexpression, mutation, or abnormal activation of TFs drives uncontrolled cell proliferation and survival. For example, oncogenic TFs such as MYC, AR, and ER are known to be constitutively active in a wide range of cancers and contribute to tumor progression and therapy resistance [1,2,3]. Therefore, the therapeutic modulation of TFs holds great potential not only for inhibiting oncogenic transcriptional programs but also for restoring normal gene expression patterns in diseased cells.

Despite their critical roles in gene regulation and disease, TFs have long been considered challenging drug targets [4]. This is due to the absence of well-defined binding pockets that conventional small-molecule inhibitors typically require. Moreover, TFs exert their functions largely through protein–protein interactions (PPIs) or protein–DNA interactions, which often involve broad, shallow, and relatively featureless interfaces that are structurally unsuitable for high-affinity small-molecule inhibitors [5]. These features have posed significant structural and technical challenges to the development of direct TF inhibitors.

**Figure 2:**
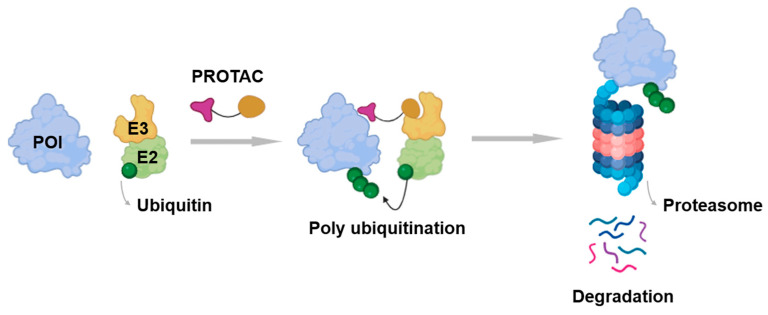
A schematic diagram of entire working mechanism of how a proteolysis-targeting chimera (PROTAC) functions as protein degrader using the UPS (ubiquitin proteasome system). The PROTAC consists of the ligand of the POI (Protein of Interest) and the ligand of E3 ligase. PThe ROTAC recruits E3 ligase to the POI and induces ubiquitination of POI, and then the POI degrades through proteasome.

An emerging approach that addresses these challenges is the development of PROTACs (proteolysis-targeting chimeras) [6]. PROTACs are bifunctional molecules that simultaneously bind a target protein and an E3 ubiquitin ligase, facilitating the ubiquitination and subsequent proteasomal degradation of the target proteins (Figure 2). Unlike traditional inhibitors that require strong binding to functional sites, PROTACs can induce degradation through weak binding without functional inhibition. This means they do not require a defined binding pocket in a functional site. This allows PROTAC technology to access previously “undruggable” targets such as TFs [7].

In parallel with academic advancements, substantial efforts from pharmaceutical and biotech companies have driven innovation in PROTAC-induced degradation of transcription factors. In addition to Arvinas, which has led the field with clinical-stage candidates such as ARV-471, companies including C4 Therapeutics, Kymera Therapeutics, Nurix Therapeutics, and Cullgen are actively developing PROTACs targeting transcription factors like STAT3, IKZF1/3, and other nuclear proteins implicated in cancer and immune disorders. These efforts underscore the therapeutic promise of overcoming the historical challenges of TF druggability through targeted protein degradation [8].

Innovative adaptations of PROTAC technology continue to expand its utility beyond conventional small-molecule applications. Strategies such as light- or enzyme-inducible PROTACs allow for spatial and temporal control of protein degradation, enabling precise manipulation of cellular pathways [9]. In addition, RNA- or aptamer-based PROTACs leverage nucleic acid-based recognition to target transcription factors or structured RNAs, offering new avenues for regulating previously undruggable targets [10]. These developments position PROTACs not only as therapeutics but also as versatile chemical biology tools for probing protein function, validating targets, and dissecting complex biological networks.

**Figure 3:**
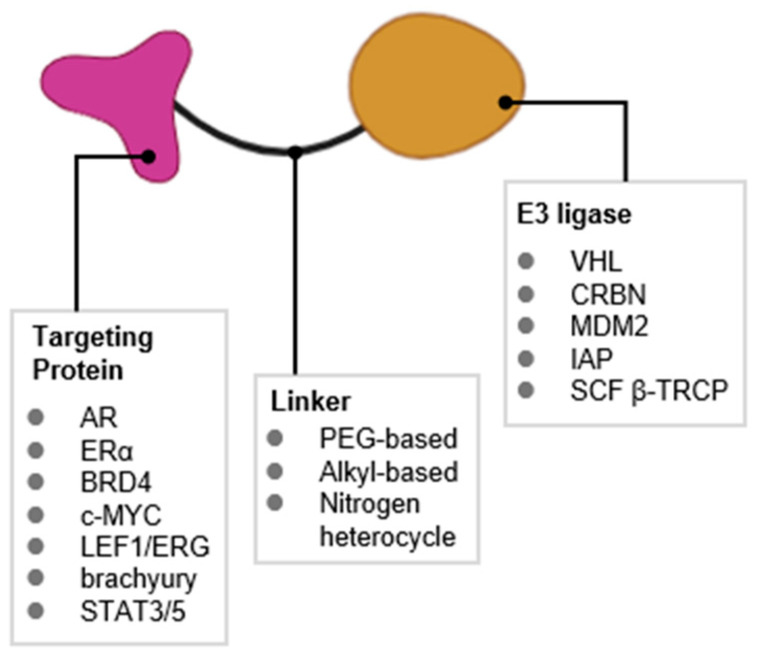
General structure and types of PROTACs targeting TFs.

Importantly, PROTACs have also been incorporated into antibody–drug conjugate (ADC) platforms as deliverable payloads to enhance tumor specificity and reduce off-target effects [11]. A representative example is BMS-986497 (formerly ORM-6151) which utilizes an anti-CD33 antibody to deliver a GSPT1-targeting PROTAC into CD33-positive tumor cells [12]. By enabling targeted degradation of GSPT1, a translation termination factor, this ADC–PROTAC conjugate disrupts oncogenic signaling with high selectivity. Such strategies illustrate the therapeutic potential of combining the targeting precision of antibodies with the catalytic, substoichiometric mechanism of PROTAC-mediated degradation, further broadening the clinical applicability of this platform. Herein, we focus on recent advances in the development of PROTACs designed to target TFs which have been traditionally considered as undruggable targets (Figure 3).

## 2. PROTACs Targeting the Androgen Receptor (AR)

The androgen receptor (AR) is a nuclear hormone receptor that is activated upon binding to testosterone or dihydrotestosterone. AR plays a pivotal role in the development and progression of prostate cancer and has therefore been extensively investigated as a therapeutic target in oncology. Given its clinical relevance, the development of PROTACs targeting AR has gained significant attention in recent years (Table 1).

The first AR-targeting PROTAC, **Protac-3**, was developed using dihydroxytestosterone (DHT) as the AR ligand and 10-mer phosphopeptide as the SCF β-TRCP E3 ligase ligand. The compound mediated SCF β-TRCP-dependent AR degradation in Hek293T cells transfected with AR-GFP plasmid at a concentration of 10 μM. Upon treatment with **Protac-3**, GFP-AP fluorescence was completely abolished in 51% of the transfected cells [13].

As an alternative approach, a peptide derived from HIF-1α (ALAPYIP), the minimum recognition domain for the VHL E3 ligase, was employed in the design of another AR-targeting PROTAC [14]. The resulting molecule, **PROTAC-5**, achieved complete degradation of GFP-AR in plasmid-transfected HEK293T cells at a concentration of 25 μM. However, due to the use of peptide-based E3 ligase ligands, these early-generation PROTACs exhibited limited cell permeability and reduced cellular potency. To address this limitation, efforts such as the incorporation of polyarginine sequences have been undertaken to improve cell penetration [15].

To enhance the pharmacokinetic properties and efficacy, a next-generation PROTAC, **ARCC-4**, was developed. This PROTAC uses enzalutamide [16], an FDA-approved AR antagonist, as the AR-binding ligand and incorporates a small-molecule ligand of VHL E3 ligase. **ARCC-4** induces VHL-mediated degradation of AR with a DC_50_ value of 5 nM in prostate cancer cell lines, effectively reducing AR protein levels [17]. Moreover, **ARCC-4** effectively degrades clinically relevant AR mutants such as F876L and T877A.

While **enzalutamide** has been a representative example as an AR-targeting ligand in PROTAC design, developing **ARI-16** [18] with superior efficacy has facilitated the advancement of PROTACs incorporating **ARI-16**-derived structures. Han et al. have utilized **ARI-16** as an AR ligand and developed AR-targeting PROTAC **ARD-69** with a DC_50_ value of less than 1 nM in AR+ prostate cancer cell lines such as LNCaP and VCaP cells. The authors optimized the structure of the VHL ligand and the linker structure to obtain optimal degradation of AR, yielding 95% inhibition of AR protein levels [19]. Moreover, **ARD-69** shows a decrease in the amount of AR proteins in the VCaP xenograft mouse model.

Additionally, **ARD-266**, which links **ARI-16** with a VHL E3 ligase ligand, was also developed by the same group [20]. Although **ARD-266** incorporates a VHL ligand with relatively weak binding affinity (Ki in the low micromolar range), it achieves potent Aryl degradation with DC_50_ values ranging from 0.2 to 1 nM in AR-positive prostate cancer cells. This finding demonstrates that effective target protein degradation can still be achieved through optimal ternary complex formation, even when using a low-affinity E3 ligase ligand.

Chen et al. designed **ARI-16** derivates as an AR ligand to have low toxicity and better binding affinity [21]. Specifically, the quaternion ring in **ARI-16** was replaced with a tropine ring and an octahydropentalene scaffold to enhance interaction with the androgen receptor. The optimized AR ligand was subsequently conjugated with a VHL E3 ligase ligand to develop a potent AR-targeting PROTAC, **A031**. The compound exhibited effective AR degradation with a DC_50_ value of 2 μM in VCaP cells. Moreover, **A031** significantly suppressed tumor growth in a zebrafish xenograft model transplanted with VCaP cells.

While most AR PROTACs utilizing **ARI-16** have been developed with VHL E3 ligase ligands, **ARD-2128** was designed to recruit the CRBN E3 ligase instead. **ARD-2128** demonstrated a highly potent DC_50_ value of 0.28 nM and showed significant inhibition of prostate cancer cell growth. Additionally, the results of daily oral administration of ARD-2128 show effective degradation of AR and exhibit great antitumor activity in the VCaP xenograft mouse model [22].

As a similar strategy, **ARD-2051** employs a further designed AR ligand with the modeling study and utilizes the CRBN E3 ligase. It demonstrates a DC_50_ value of 0.6 nM and Dmax greater than 90% in LNCaP and VCaP cell lines. Moreover, **ARD-2051** shows favorable oral bioavailability and significant antitumor activity in the VCaP xenograft mouse model [23].

Within the same research group, another PROTAC, **ARD-1676**, was reported, which incorporates the AR ligand from ARD-2051 and a novel CRBN E3 ligase ligand [24]. **ARD-1676** shows remarkable AR degradation with DC_50_ values of 0.1 and 1.1 nM in AR+ VCaP and LNCaP cell lines, respectively. Additionally, **ARD-1676** effectively induces degradation of clinically relevant AR mutant. Oral administration of ARD-1676 effectively inhibits tumor growth in the VCaP xenograft mouse model.

Another potent AR ligand, developed by scientists at Pfizer [18], has been employed in the design of the AR-targeting PROTAC **ARD-2585**. The AR ligand has been used in **ARV-110**, which was the representative AR-targeting PROTAC in clinical trial phase 1/2. **ARD-2585** demonstrates exceptional potency with a DC_50_ value of less than 0.1 nM in VCaP and LNCaP cells. Furthermore, it exhibits favorable pharmacokinetic properties and effective inhibition of VCaP tumor growth in the xenograft mouse model [25].

While most AR-targeting PROTACs utilize AR ligands binding in the C-terminal ligand-binding domain (LBD), Zhang et al. reported the first PROTAC targeting the N-terminal domain (NTD) of AR, resulting in the development of **BWA-522**. The authors employed NTD inhibitor ralaniten as an AR ligand and a CRBN E3 ligase ligand in their AR-targeting PROTAC design. **BWA-522** exhibited AR degradation with a DC_50_ value of 3.5 μM in LNCaP cells and demonstrated effective tumor growth inhibition following oral administration [26].

Currently, **ARV-110** and AR-targeting PROTACs developed by Arvinas, are being evaluated in phase 1/2 clinical trials, highlighting the potential of PROTACs to effectively modulate challenging target proteins [27,28].

**Table 1:** AR (androgen receptor)-targeting PROTACs.

PROTAC	E3 Ligase	Target Protein Binder	Structure	DC_50_	Ref.
**Protac-3**	SCF β-TRCP	DHT	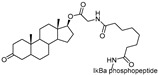	10 μM	[13]
**PROTAC-5**	VHL	DHT	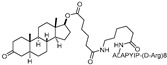	-	[15]
**ARCC-4**	VHL	Enzalutamide	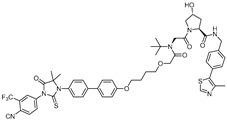	5 nM	[17]
**ARD-69**	VHL	ARI-16	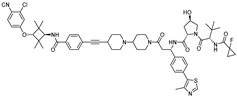	~1 nM	[19]
**ARD-266**	VHL	ARI-16	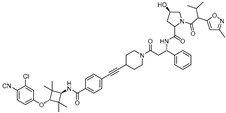	0.5 nM	[20]
**A031**	VHL	ARI-16-derivatives	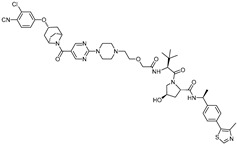	2 μM	[21]
**ARD-2128**	CRBN	ARI-16	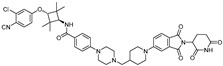	0.28 nM	[22]
**ARD-2051**	CRBN	AR 4034	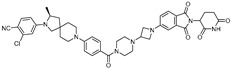	0.6 nM	[23]
**ARD-1676**	CRBN	AR 4034	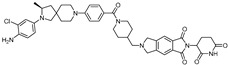	1.1 nM	[24]
**ARD-2585**	CRBN	AR antagonist	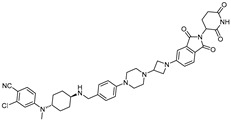	0.04–0.1 nM	[25]
**BWA-522**	CRBN	Ralaniten	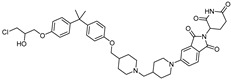	0.73 μM	[26]
**ARV-110**	CRBN	AR ligand-30	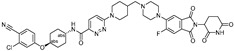	10 nM	[27,28]
**ARV-766**	CRBN		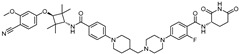	<1 nM	

## 3. PROTACs Targeting Bromodomain-Containing Protein 4 (BRD4)

BRD4 is a member of the BET (Extra-Terminal motif) protein family that modulates gene transcription by recognizing acetylated histones and promoting the expression of genes involved in cell cycle progression and cell survival. Dysregulation of BRD4 has been implicated in various diseases including cancer, inflammatory disorders, and fibrosis [29], making it an important therapeutic target. Given the significance of BRD4 as a therapeutic target, small-molecule inhibitors such as **JQ-1**, **I-BET762**, and **OTX015** have been developed to block its activity [30]. While the developed inhibitors effectively repress the binding of BRD4 to chromatin, they do not disrupt other functional roles of BRD4 such as non-bromodomain-dependent interactions. To fully disrupt the functional role of BRD4, the complete degradation of BRD4 using PROTACs has been widely studied (Table 2).

With the discovery of **JQ-1** as an effective BRD4 inhibitor, several PROTACs utilizing **JQ-1** have been developed. **dBET1** represents the first PROTAC developed to directly target BRD4, utilizing a combination of the BRD4 inhibitor **JQ-1** and a CRBN E3 ligase ligand, phthalimide [31]. It induces CRBN-dependent degradation of BET family proteins, including BRD2, BRD3, and BRD4, in human acute myeloid leukemia (AML) cells with a DC_50_ of 100 nM. Notably, **dBET1** demonstrates an enhanced apoptotic response and an inhibitory effect on proliferation in AML cells compared to the BET inhibitor **JQ-1**.

Subsequently, **MZ1** emerged as another PROTAC targeting BRD4 with improved efficacy and selectivity among BET family proteins [32]. **MZ1**, composed of a VHL E3 ligase ligand and **JQ-1**, induces degradation of BRD4 at concentrations as low as 100 nM in AML cells, whereas other BET proteins require concentrations around 1 µM for degradation. Notably, **MZ1** remains stable at low concentrations, minimizing its competition with endogenous VHL substrates and avoiding unintended stabilization of HIF-1α. **MZ1** represents the first PROTAC specifically designed for selective BRD4 degradation.

While most PROTACs have been developed using CRBN or VHL E3 ligase ligands, ligands of other E3 ligases such as MDM2, IAP, and UBR have been identified [33,34,35]. However, their application in PROTAC design remains limited. Hines et al. reported a BRD4-targeting PROTAC, **A-1874**, which connects the MDM2 E3 ligase ligand Nutlin with JQ-1 [36]. The compound demonstrated robust protein degradation up to 98% at 100 nM concentrations and improved anticancer efficacy compared to previously reported BRD4 PROTACs across various cancer cell types, including colorectal cancer, melanoma, and lung cancer cells. Notably, since Nutlin stabilizes the tumor suppressor protein p53 [37], it is considered to have complementary activity to the protein degradation effect of **A-1874** [38]. In cell lines lacking functional p53, **A-1874** exhibited significantly reduced efficacy in decreasing cell viability compared to wild-type p53 cell lines, indicating that Nutlin-based PROTACs can selectively target cells based on p53 status.

Although **JQ-1** has shown high potency in BRD4 inhibition, the compound has not been tried in clinical trials due to its instability and poor bioavailability. A structurally modified derivative, **OTX015**, was developed for clinical trials by OncoEthix with improved drug-like properties. Due to the improved pharmacokinetics of **OTX015**, a PROTAC utilizing this compound has also been developed. **ARV-825**, composed of **OTX015** and the CRBN E3 ligase ligand, achieves a DC_50_ value of 4.75 nM against BRD4 and effectively suppresses tumor growth [39]. The compound induces significant downstream c-MYC suppression and robust apoptosis induction in Burkitt’s lymphoma (BL) cells compared to the known BRD4 inhibitors, **JQ-1** and **OTX015**.

Another BET inhibitor in clinical trials, **ABBV-075** [40], has been used for the development of a selective BRD4-targeting PROTAC known as compound **6b**. It induces CRBN-mediated proteasomal degradation of BRD4 and achieves robust degradation at 100 nM in basal-like breast cancer cell lines. Notably, **6b** selectively degrades BRD4 without affecting BRD2 or BRD3, highlighting its specificity conferred by the **ABBV-075** scaffold [41]. It also represses the expression of Krüppel-like factor 5 (KLF5), a downstream target of BRD4, resulting in a robust inhibitory effect on tumor growth in the xenograft mouse model.

**Table 2:** BRD4 (bromodomain 4)-targeting PROTACs.

PROTAC	E3 Ligase	Target Protein Binder	Structure	DC_50_	Ref.
**dBET1**	CRBN	JQ-1	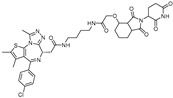	~100 nM	[31]
**MZ1**	VHL	JQ-1	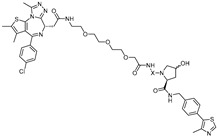	~1 μM	[32]
**A-1874**	MDM2	JQ-1	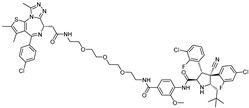	-	[36]
**ARV-825**	CRBN	OTX-015	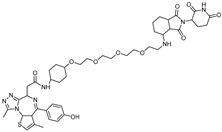	~1 nM	[39]
**6b**	CRBN	ABBV-075	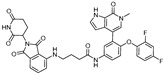	~10 nM	[41]

## 4. PROTACs Targeting ERα

ERα is one of the most frequently targeted proteins by PROTAC technology, as it is a well-known oncogenic transcription factor implicated in the development of breast cancer [42]. In this section, we introduce studies that have employed PROTACs to induce the degradation of ERα (Table 3).

The first proof-of-concept study demonstrating an ERα-targeting PROTAC was reported by Sakamoto et al., in which an estradiol-based PROTAC, **PROTAC-2**, was designed to recruit ERα to the SCF β-TRCP E3 ligase [13]. This chimeric molecule consisted of a phosphopeptide ligand for β-TRCP linked to estradiol (E2), enabling the proteasomal degradation of ERα at 1 μM in vitro. This demonstrated the possibility of PROTAC technology as a potential therapeutic strategy in targeting TFs.

Subsequently, Zhang et al. developed another ERα-targeting PROTAC, **E2-SMPI**, which utilized a HIF-1α-derived octapeptide to recruit VHL E3 ligase and E2 as the ERα ligand [43]. The HIF-1α-derived octapeptide, a 10-mer sequence, facilitated recognition by VHL, and the developed PROTAC induced ERα degradation with a DC_50_ value of 50 μM in MCF-7 cells. Although the relatively high DC_50_ indicates limited potency and poses a challenge for in vivo application, this study primarily served as a proof-of-concept to demonstrate the feasibility of peptide-based ERα degradation. Further optimization of the E3 ligase binding peptide and linker components in PROTAC has been pursued to improve its efficacy [15,44,45]. However, due to the inherent limitations of peptide-based molecules, such as low stability and poor cell permeability, the development of ERα-targeting PROTACs has shifted toward small-molecule-based approaches.

Upon the identification of a small-molecule ligand to replace the peptide-based VHL E3 ligase ligand, the first ERα-targeting PROTAC utilizing small-molecule VHL E3 ligase ligand, **ERD-308**, was subsequently developed and reported [46]. **ERD-308** employed Raloxifene, a highly potent selective estrogen receptor modulator, and exhibited robust ERα degradation with a DC_50_ value of 0.17 nM in MCF-7 cells. Moreover, it induced >95% ERα degradation at 5 nM.

As an alternative approach, Xie et al. utilized a novel ERα pharmacophore skeleton, oxabicycloheptane sulfonamide (OBHSA) [47] scaffold, as an ERα ligand in PROTAC design. The resulting PROTAC, **ZD12**, showed potent antiproliferative effects and complete ERα degradation even at 10 nM in a broad spectrum of ERα+ breast cancer cells, including tamoxifen-resistant and ERα mutant cell lines. Furthermore, **ZD12** effectively suppressed tumor growth in drug-resistant breast cancer mouse models [48].

In 2024, Wang et al. reported fluorescent probe-based ERα PROTAC **Compound A3**, enabling the real-time monitoring with targeted protein degradation [49]. **Compound A3** exhibited potent ERα degradation (DC_50_ = 0.12 μM) mediated with VHL E3 ligase, strong anti-proliferative activity (IC_50_ = 0.051 μM), and favorable fluorescence properties suitable for live-cell tracking. This work highlights the emerging potential of dual-function PROTACs as tools for both therapeutic strategy and dynamic visualization of degradation processes.

While various E3 ligases such as CRBN, VHL, and MDM2 have been utilized in PROTAC design, inhibitors of apoptosis proteins (IAPs) have also been employed to develop IAP-based PROTACs, known as specific and non-genetic IAP-dependent protein erasers (SNIPERs) [50,51]. The first ERα-targeting SNIPERs used a natural ERα agonist, estrone, in combination with a cellular IAP (cIAP) ligand, resulting in a compound (**SNIPER(ER)-11**) [52]. The compound completely degraded ERα at 30 μM in ERα-positive cells.

To enhance in vivo efficacy, Ohoka et al. reported on **SNIPER(ER)-87**, which incorporates 4-hydroxytamoxifen (4-OHT) as the ERα ligand and **LCL161** as the ligand for X-linked IAP (XIAP). Unlike the earlier SNIPERs that used bestatin, a modest-affinity cIAP ligand, **SNIPER(ER)-87** achieved potent ERα degradation with a DC_50_ value of 3 nM in MCF-7 cells and significantly suppressed ERα-positive breast tumor growth in mice. Further structure–activity relationship (SAR) studies have also been conducted to optimize ERα-targeting SNIPERs [53,54].

Given that TFs function through sequence-specific DNA recognition, recently, PROTACs utilizing an oligonucleotide as the target TFs’ ligand have been explored to selectively degrade TFs. In 2022, Zhang et al. reported a double-stranded DNA (dsDNA)-conjugated PROTAC, **ERE-PROTAC**, utilizing a natural estrogen response element (ERE) sequence as an ERα ligand [55]. The dsDNA was conjugated to the VHL E3 ligase ligand via a click reaction, resulting in effective ERα degradation with a DC_50_ value around 5 μM and cell death in ERα+ breast cancer cells.

Using a similar strategy, Miyako et al. developed **LCL-ER(dec)**, a dsDNA-conjugated PROTAC containing the ERα binding sequence and cIAP E3 ligase ligand [56]. The dsDNA used as the ERα ligand has 13-mer-truncated sequence derived from an ERE sequence, resulting in 21-mer dsDNA. **LCL-ER(dec)** effectively induced ERα degradation at a concentration of 10 μM and suppressed estrogen-dependent transcriptional activity of ERα in MCF-7 cells.

In addition, the ERα-binding aptamer **ER(apt)D1** [57], previously identified through SELEX, was utilized in the PROTAC design. The aptamer was conjugated to a CRBN E3 ligase ligand, resulting in the development of an aptamer-based ERα-targeting PROTAC, **POM-ER(apt)D1**. This compound induces ERα degradation with a DC_50_ value of approximately 5 μM and inhibits ERα-mediated transcriptional activity in MCF-7 cells [58].

With the continuous efforts to develop PROTACs targeting ERα, **ARV-471**, a PROTAC developed collaboratively by Arvinas and Pfizer, is currently being evaluated in phase 3 clinical trials. **ARV-471** has shown promising efficacy in effectively degrading the estrogen receptor and reducing tumor size, both as a monotherapy and in combination with CDK4/6 inhibitors [59,60]. This represents a compelling example of how PROTAC technology can enable drug development against targets previously considered undruggable.

**Table 3:** ERα (estrogen receptor alpha)-targeting PROTACs.

PROTAC	E3 Ligase	Target Protein Binder	Structure	DC_50_	Ref.
**PROTAC-2**	SCF β-TRCP	E2	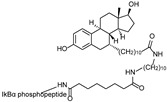	-	[13]
**E2-SMPI**	VHL	E2	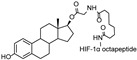	50 µM	[43]
**ERD-308**	VHL	Raloxifene	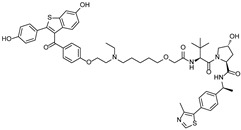	0.17 nM	[46]
**ZD12**	VHL	OBSHA ligand	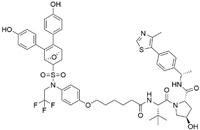	-	[48]
**Compound A3**	VHL	highly ER targeting NIR fluorescent probe	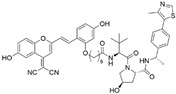	0.12 µM	[49]
**SNIPER(ER)-11**	cIAP	estrone	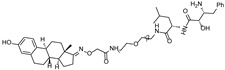	-	[52]
**SNIPER(ER)-87**	IAP	4-hydroxyl tamoxifen	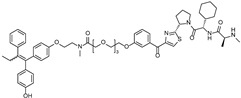	3 nM	[53,54]
**ERE-PROTAC**	VHL	GTCCAAAGTCAGGTCA-CAGTGACCTGATCAAAGT- (ds)	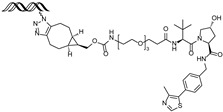	~5 µM	[55]
**LCL-ER(dec)**	IAP	GTCAGGTCACAGTGACCTGAT (ds)	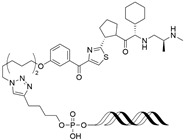	~3 µM	[56]
**POM-ER(apt)D1**	POM	nucleic acid aptamers	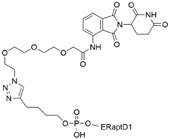	~5 µM	[58]
**ARV-471**	CRBN		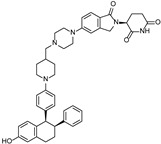	0.9 nM	[59,60]

## 5. PROTACs Targeting Other TFs

While most PROTACs targeting transcription factors have focused on AR, ER, and BRD4, recent studies have reported PROTACs targeting a wider range of transcription factors (Table 4). These PROTACs utilize either DNA sequences, as exemplified by ERα-targeting PROTACs, or small-molecule ligands that directly bind to transcription factors, achieving high specificity and efficacy against traditionally undruggable targets.

By leveraging the ability of transcription factors to bind to specific DNA sequences, Shao et al. have reported oligonucleotide-based PROTACs (O’PROTACs), which utilize double-stranded oligonucleotides as the POI binder. The authors developed O’PROTACs targeting lymphoid enhancer-binding factor 1 (LEF1) and the ETS-related gene (ERG), two transcription factors highly implicated in cancer. The LEF1-targeting O’PROTAC (**LEF1 OP-V1**) and the ERG-targeting O’PROTAC (**ERG OP-C-N1**) both employed a VHL E3 ligase ligand, effectively inducing target protein degradation in nanomolar concentrations in the tens and subsequently inhibiting cancer cell growth both in vitro and in vivo [61].

Samarasinghe et al. reported O’PROTACs targeting other oncogenic transcription factors, c-Myc and Brachyury. These transcription factors have been considered as undruggable targets due to the shallow surface without binding pockets. Double-stranded oligonucleotides which are c-Myc or Brachyury binding sequences have been conjugated with the VHL ligand using a click reaction, resulting in **OT3** and **OT7** [62]. Both compounds effectively degrade c-Myc or Brachyury, respectively, with a DC_50_ value of 50 nM, suppressing tumorigenesis. Inspired by this approach, **MP-16** and **MP-17** were generated by conjugating the VHL E3 ligase ligand to a rationally optimized and truncated double-stranded DNA sequence specific for the c-Myc complex [63]. These constructs exhibited efficient c-Myc degradation and robust antitumor efficacy in hepatocellular carcinoma models.

Other than binding ds-oligonucleotides, modified oligonucleotides or aptamers were utilized in PROTACs targeting c-Myc. Li et al. introduced a novel PROTAC called **TEP**, constructed from threose nucleic acid (TNA) and DNA, which led to efficient degradation of c-Myc. This approach not only suppressed the growth of triple-negative breast cancer (TNBC) cells but also improved their responsiveness to the CDK inhibitor **palbociclib** [64]. Through an alternative strategy, Wang et al. designed an aptamer-based PROTAC, **circPA1-ProMyc**, by linking a c-Myc-targeting single-stranded DNA aptamer with an anti-PD-L1 aptamer to enhance drug delivery. This dual-targeting strategy enabled the concurrent degradation of c-Myc and Max proteins [65].

For small-molecule-based PROTACs targeting TFs, a prominent example is **SD-36**, a PROTAC designed to degrade signal transducer and activator of transcription 3 (STAT3), a key factor in tumor progression [66]. By linking a STAT3 SH2 domain inhibitor (**SI-109**) to a CRBN E3 ligase ligand, **SD-36** induces rapid and selective degradation of STAT3 at nanomolar concentrations with minimal effect on other STAT proteins. It shows strong antiproliferative effects in STAT3-driven leukemia and lymphoma models and leads to complete tumor regression in xenograft mice.

Similarly, **AK-2292** was developed and selectively degraded both signal transducer and activator of transcription 5A (STAT5A) and 5B (STAT5B), while sparing other STAT family proteins and thousands of unrelated proteins [67]. It effectively inhibits STAT5 signaling in a sub-micromolar concentration and induces tumor regression in chronic myeloid leukemia (CML) xenograft models with good tolerability. **AK-2292** serves as both a useful biological tool and a promising lead for STAT5-targeted cancer therapy.

Beyond oncology, a VHL-recruiting PROTAC was designed to degrade SMAD family member 3 (Smad3), a key mediator of fibrosis, while stabilizing hypoxia-inducible factor 2 alpha (HIF-2α), a protective factor in kidney disease. By conjugating Smad3- and VHL E3 ligase-binding small molecules, the optimized PROTAC effectively degraded Smad3 and stabilized HIF-2α in both in vitro and in vivo models. It highlights the potential for expanding PROTAC applications into chronic, non-cancer diseases [68].

Collectively, these PROTACs demonstrate the expanding potential of targeted protein degradation strategies not only against transcription factors but also across a broader spectrum of disease targets. They suggest the feasibility of modulating previously undruggable proteins, thereby reinforcing the promise of PROTACs as a next-generation therapeutic modality.

**Table 4:** Other transcription-factor-targeting PROTACs.

PROTAC	Target Protein	E3 Ligase	Target Protein Binder	Structure	DC_50_	Ref.
**LEF1 OP-V1**	LEF1	VHL	TACAAAGATCAAAGGGTT (ds)	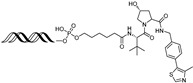	25 nM	[61]
**ERG OP-C-N1**	ERG	VHL	ACGGACCGGAAATCCGGTT (ds)	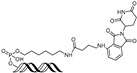	182.4 nM	[61]
**OT17**	brachyury	VHL	TCCAATTTCACACCTAGGTGTGAAATTGGG (ds)	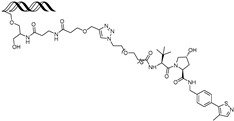	75 nM	[62]
**OT7**	c-MYC	VHL	GAGCACGTGGTTGCCACGTGGTT (ds)	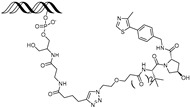	50 nM	[62]
**MP-16**	c-MYC	VHL	GAGTAGCACGTGCTAC (ds)	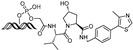	~0.8 µM	[63]
**MP-17**	c-MYC	VHL	GAGTAGCACGTGCTAC (ds)	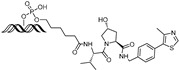	~0.8 µM	[63]
**TEP**	c-MYC	pomalidomide	nucleic acid aptamers	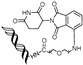	~100 nM	[64]
**circPAI-ProMYC**	c-MYC	CRBN	nucleic acid aptamers	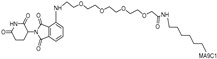	5.02 nM	[65]
**SD-36**	STAT3	CRBN	**SI-109**	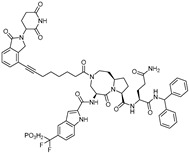	0.06 µM	[66]
**AK-2292**	STAT5	CRBN	STAT5 ligand	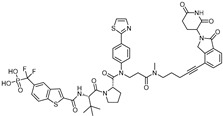	~1 µM	[67]

## 6. Conclusions

In conclusion, PROTAC technology has emerged as a promising therapeutic strategy for targeting disease-associated TFs. Although TFs have traditionally been considered challenging targets due to their broad, shallow binding surfaces, recent advances have demonstrated successful targeting of these proteins using known inhibitors or ligands as warheads in PROTAC design.

While further studies and clinical trials are needed to fully elucidate the therapeutic efficacy of TF-targeting PROTACs, the early findings are highly encouraging. To fully harness the potential of PROTACs in transcription factor modulation, it is critical to expand the repertoire of E3 ligases and target proteins, as well as to optimize pharmacokinetic properties. Broadening the diversity of usable E3 ligases may enable the degradation of more diverse TFs.

As PROTACs continue to advance, this technology will offer an innovative approach to targeting previously “undruggable” transcription factors, ultimately improving therapeutic outcomes for patients.

## Abbreviations

The following abbreviations are used in this manuscript:

TFs	Transcription factors
PPIs	Protein–protein interactions
PROTAC	Proteolysis-targeting chimeras
AR	Androgen receptor
ERα	Estrogen receptor alpha
BRD4	Bromodomain 4
VHL	Von Hippel-Lindau
DC_50_	Half-maximal degradation concentration
CRBN	Cereblon
MDM2	Murine double minute 2
DHT	Dihydroxytestosterone
LBD	Ligand-binding domain
NTD	N-terminal domain
BET	Extra-Terminal motif
AML	Acute myeloid leukemia
KLF5	Krüppel-like factor 5
BL	Burkitt’s lymphoma
OBHSA	Oxabicycloheptane sulfonamide
IAP	Inhibitors of apoptosis proteins
SNIPER	Specific and non-genetic IAP-dependent protein eraser
cIAP	Cellular IAP
4-OHT	4-hydroxytamoxifen
XIAP	X-linked IAP
SAR	Structure–activity relationship
dsDNA	Double-stranded DNA
ERE	Estrogen response element
O’PROTAC	Oligonucleotide-based PROTAC
LEF1	Lymphoid enhancer-binding factor 1
ERG	ETS-related gene
TNA	Threose nucleic acid
TNBC	Triple-negative breast cancer
STAT3	Signal transducer and activator of transcription 3
STAT5	Signal transducer and activator of transcription 5
CML	Chronic myeloid leukemia
Smad3	SMAD family member 3
HIF-2α	Hypoxia-inducible factor 2 alpha
